# Uncovering the Bone-Muscle Interaction and Its Implications for the Health and Function of Older Adults (the Wellderly Project): Protocol for a Randomized Controlled Crossover Trial

**DOI:** 10.2196/18777

**Published:** 2021-04-09

**Authors:** Cassandra Smith, Xuzhu Lin, David Scott, Tara C Brennan-Speranza, Ahmed Al Saedi, Alba Moreno-Asso, Mary Woessner, Ebrahim Bani Hassan, Nir Eynon, Gustavo Duque, Itamar Levinger

**Affiliations:** 1 Institute for Health and Sport Victoria University Melbourne VIC Australia; 2 Australian Institute for Musculoskeletal Science University of Melbourne and Western Health Melbourne VIC Australia; 3 Institute for Physical Activity and Nutrition School of Exercise and Nutrition Sciences Deakin University Geelong, VIC Australia; 4 Department of Medicine, School of Clinical Sciences at Monash Health Monash University Clayton VIC Australia; 5 School of Medical Sciences and School of Public Health, Faculty of Medicine and Health University of Sydney New South Wales Australia; 6 Department of Medicine-Western Health The University of Melbourne Melbourne, VIC Australia; 7 Institute for Health and Sport Victoria University Melbourne, VIC Australia

**Keywords:** acute exercise, clinical trial, bone, adult, aging, osteocalcin, muscles, sarcopenia, progenitor cells, stem cells

## Abstract

**Background:**

Bone and muscle are closely linked anatomically, biochemically, and metabolically. Acute exercise affects both bone and muscle, implying a crosstalk between the two systems. However, how these two systems communicate is still largely unknown. We will explore the role of undercarboxylated osteocalcin (ucOC) in this crosstalk. ucOC is involved in glucose metabolism and has a potential role in muscle maintenance and metabolism.

**Objective:**

The proposed trial will determine if circulating ucOC levels in older adults at baseline and following acute exercise are associated with parameters of muscle function and if the ucOC response to exercise varies between older adults with low muscle quality and those with normal or high muscle quality.

**Methods:**

A total of 54 men and women aged 60 years or older with no history of diabetes and warfarin and vitamin K use will be recruited. Screening tests will be performed, including those for functional, anthropometric, and clinical presentation. On the basis of muscle quality, a combined equation of lean mass (leg appendicular skeletal muscle mass in kg) and strength (leg press; one-repetition maximum), participants will be stratified into a high or low muscle function group and randomized into the controlled crossover acute intervention. Three visits will be performed approximately 7 days apart, and acute aerobic exercise, acute resistance exercise, and a control session (rest) will be completed in any order. Our primary outcome for this study is the effect of acute exercise on ucOC in older adults with low muscle function and those with high muscle function.

**Results:**

The trial is active and ongoing. Recruitment began in February 2018, and 38 participants have completed the study as of May 26, 2019.

**Conclusions:**

This study will provide novel insights into bone and muscle crosstalk in older adults, potentially identifying new clinical biomarkers and mechanistic targets for drug treatments for sarcopenia and other related musculoskeletal conditions.

**Trial Registration:**

Australia New Zealand Clinical Trials Registry ACTRN12618001756213; https://www.anzctr.org.au/Trial/Registration/TrialReview.aspx?id=375925.

**International Registered Report Identifier (IRRID):**

DERR1-10.2196/18777

## Introduction

### Background

Adults reach their peak muscle and bone mass in the third decade of life, after which an age-related loss of skeletal muscle and bone mass occurs [1,2]. Under certain conditions, for reasons that are not fully understood, this loss of bone mass (osteoporosis) and muscle (sarcopenia) is accelerated and, in some cases, occurs concurrently [3-5]. Emerging evidence suggests that this parallel and exponential loss of bone and muscle mass and strength is driven, at least in part, by bone and muscle crosstalk. The skeleton and skeletal muscle are closely linked anatomically, biochemically, and metabolically and modulate each other in endocrine and paracrine manners [6]. Many factors may be involved in this crosstalk, including genetics, changes in vitamin D and parathyroid hormone (PTH) levels, aging, increased levels of systemic and local inflammatory markers (ie, interleukin-6 [IL-6] and tumor necrosis factor), obesity and adipokines, mechanical loading, and altered hormones (ie, osteocalcin, resistin, and myostatin) [7,8]*.* The exact mechanisms involved in this crosstalk remain partially explored, although it has been proposed that undercarboxylated osteocalcin (ucOC) and possibly circulating osteoprogenitor (COP) cells may be mediators [6,9-12].

Serum total osteocalcin (tOC) is an osteoblast-specific secreted protein within the circulation that can be present in two major forms, as follows: γ-carboxylated osteocalcin (cOC) and ucOC lacking γ-carboxylation at one or more sites [13]. cOC, which is predominantly located in bone, is at least partly involved in bone mineralization, whereas ucOC has been shown to be involved in glucose metabolism—at least in mice—with new evidence suggesting a role in influencing muscle mass and strength [10,14-24]. Osteocalcin-deficient mice have reduced muscle mass and strength [17], and lower ucOC levels following hindlimb immobilization in rats are associated with reduced muscle mass and muscle force [25]. Treatment with ucOC can increase the cross-sectional area of the extensor digitorum longus, improve grip strength in mice, and stimulate myotube formation in C2C12 myoblast cultures in vitro [16]. In humans, we and others have shown that exercise increases serum ucOC levels and improves muscle metabolism and whole-body glucose control [10], most likely via increased insulin signaling protein levels within skeletal muscle, and that a decreased ratio of ucOC/tOC correlates with lower muscle strength in older women [19]. However, the effect of acute exercise on ucOC in older adults remains unknown; in particular, the role of ucOC in human myotubes and its association with muscle function parameters (ie, strength and mass) remain unclear.

Exercise causes a series of physiological responses in the bone and skeletal muscle, improving glucose regulation and insulin sensitivity and, importantly, promoting pro-osteogenic factors, including increasing bone formation biomarkers such as osteocalcin [26-31]. Exercise is a known nonpharmacological approach to improving bone health, reducing the risk of osteoporosis, and, importantly, concomitantly improving muscle function [32-35]. Thus, exercise represents an efficacious approach in older persons to reduce age-associated alterations related to sarcopenia, which currently has no available drug treatments. Evidence suggests that various mechanical factors including exercise stimulate the differentiation of mesenchymal stem cells (MSCs) into osteoblasts [36,37]. COP cells circulate within the blood and are MSC-like with osteogenic potential and a precursor for the osteoblastic lineage and potentially osteocalcin [11]. It remains unknown whether exercise, with stimuli promoting pro-osteogenic factors (ie, high load bearing resistance exercise [RE], impact, and jumping exercise), can mitigate the aging process in skeletal muscle and bone through its effect on COP cell levels and therefore osteocalcin [38]. Even a single acute bout of exercise (ie, aerobic exercises [AERs] and REs) elicits positive effects on the bone endocrine and biomarker response and can increase ucOC and insulin sensitivity [9,10,39-42]. However, the exact mechanism by which ucOC influences skeletal muscle function, strength, and metabolism in older adults remains unclear.

### Objectives

The primary objective of this study is to determine the change in circulating ucOC following acute exercise interventions between older adults with low and high muscle function (stratified based on leg muscle quality [LMQ]; see the *Lower Limb Maximal Strength and LMQ* section for the LMQ equation). To extend the current knowledge of ucOC in humans, and as an adjunct to this study, we will also perform in vitro experiments on cultured primary myotubes to uncover the mechanistic pathways of action of ucOC. In addition, as a secondary objective, we will aim to quantify the lineage of COP cells before and after exercise, as COP cells can potentially act as a regeneration and antiaging inducible factor and a precursor for osteocalcin.

### Hypotheses

We hypothesize that older adults with lower muscle function, compared to those with normal or higher muscle function, will (1) be characterized by lower levels of circulating ucOC and those with lower ucOC will be associated with poorer glucose control and (2) be characterized by abnormal skeletal muscle signaling (muscle hypertrophic or atrophic pathways). Both AER and RE will increase ucOC; however, we hypothesize that this will be to a greater degree in those with lower muscle function. We will test these hypotheses at baseline and after acute exercise. This project has the potential to identify novel biomarkers for interactions between bone and muscle, with implications for future drug targets or clinical interventions and the management of those with or at risk of sarcopenia or reduced muscle function.

## Methods

### Design

**Figure 1:**
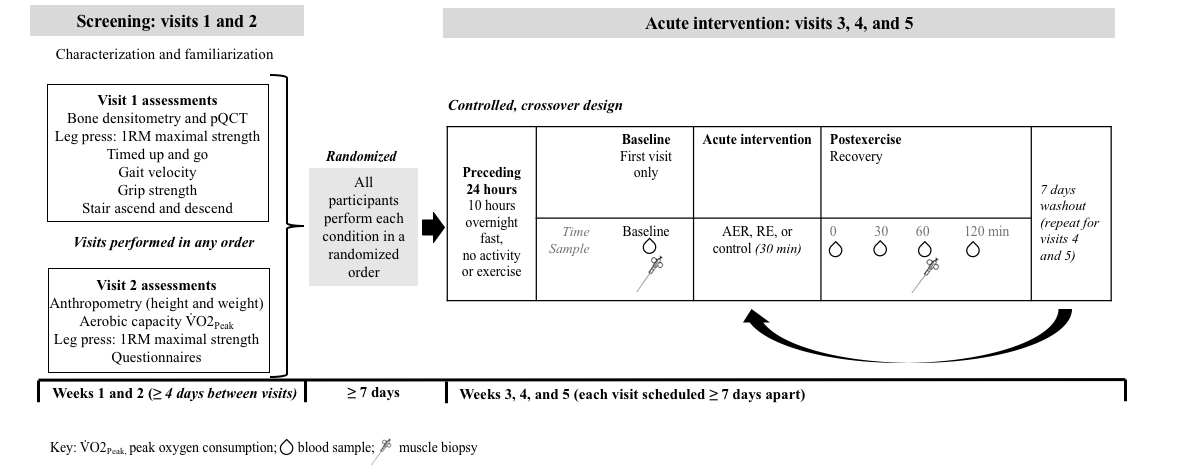
Study design. 1RM: one-repetition maximum; AER: aerobic exercise; pQCT: peripheral quantitative computed tomography; RE: resistance exercise.

This study is a randomized controlled crossover trial (Figure 1) approved by the Melbourne Health (MH) Human Research Ethics Committee (reference number: 2017/08) and is registered with the Australian New Zealand Clinical Trials Registry (trial number: ACTRN12618001756213). The trial is a multicenter clinical trial conducted at the Institute for Health and Sport, Victoria University, Melbourne, Victoria, Australia, and the Australian Institute for Musculoskeletal Science (AIMSS) in Western Health, St Albans, Victoria, Australia. The trial will be conducted in accordance with the Helsinki Declaration, and reporting of the study will adhere to the CONSORT (Consolidates Standards of Reporting Trials) guidelines [43,44].

### Participants

Men and women aged 60 years or older will be recruited. Women will be required to be a minimum of 12 months postmenopause; this is because of the potential alteration in hormones that occur during perimenopause, which can interact with or affect the specific project outcomes of this study. The inclusion and exclusion criteria are listed in Textbox 1. Additional study exclusions will include the inability to provide informed consent independently for safety reasons (particularly as we take some invasive measures) and an inability to understand English, as this may potentially be a safety concern if unable to communicate during visits that include maximal exertion testing and acute exercise bouts.

**Study eligibility.**

**Inclusion criteria**

- Males and females aged 60 years
- Females >12 months postmenopause

**Exclusion criteria**

- Any fractures within the previous 3 months
- Have begun a new osteoporotic treatment within the previous <3 months or have begun taking antiresorptive medications within the previous <3 months
- Have diabetes mellitus or are taking hyperglycemic medications
- Any hematological, myelodysplastic, or myeloproliferative disorder
- Any bone malignancy
- Taking warfarin of vitamin K supplementation or restriction
- BMI≥40 kg/m^2^
- Engagement in a resistance exercise regime for more than 2 sessions per week

### Recruitment

Prospective participants will be recruited using advertisement flyers. These will be displayed at Western Health sites (Sunshine and Footscray Hospitals, Victoria, Australia) and provided for use within the general community and other media outlets. Interested participants will self-initiate contact with the research team via email or phone. Those interested will be screened against the inclusion and exclusion criteria. Eligible participants will be provided with information for the participants and participant-informed consent forms. A physical examination and an approval to participate in the study will be required from the patients’ physician. Please refer to Multimedia Appendix 1 for the schedule of enrollment, interventions, and assessments.

### Initial Screening

#### Summary of Initial Screening

The initial screening will be used for clinical characterization of the volunteers as well as for bone and muscle quantification and quality. It includes 2 separate visits of 3-4 hours’ duration (visits 1 and 2; Figure 1), performed in any order and up to 14 days apart. Both visits will be performed in the morning and following an overnight fast. The measures obtained during these visits are explained in detail below.

#### Bone and Muscle Health

##### Dual Energy X-Ray Absorptiometry

Body composition and bone mineral density (BMD) will be assessed using a dual energy x-ray absorptiometry (DXA) scanner (Hologic, Horizon A, software version 5.6.0.4). Total BMD as well as the neck of the femur and lumbar spine BMD will be assessed. Lean body mass and fat mass will also be assessed. The DXA scan will ideally be performed in the morning following an overnight fast by experienced personnel. This will be completed by the AIMSS.

##### Bone Microarchitecture and Fat Infiltration

Peripheral quantitative computed tomography (pQCT; Stratec XCT3000, Stratec Medizintechnik GmbH) will be used to quantify muscle and bone mass, density, and adipose infiltration at the nondominant forearm and foreleg [45,46].

Single 2.5-mm transverse scans will be obtained at 4% and 66% of tibial length (measured form the palpable tip of medial malleolus) and 4% and 66% of the radial length (from the radial condyle), with a voxel size of 0.4 mm. All pQCT scans will be acquired and analyzed by an experienced operator, and the device will be calibrated on the scan date using the manufacturer’s phantom. Calf and forearm muscle cross-sectional areas (mm^2^) and densities (mg/cm^3^) will be determined using the manufacturer’s algorithms and software (version 6.2). The calf intramuscular adipose tissue cross-sectional area (cm^2^) will be quantified as previously described [47]. Trabecular and cortical bone densities and structure will be assessed at the relevant regions of interest. All imaging will be performed by an appropriate expert (radiographer).

#### Blood Sample

##### Quantification of Bone Remodeling and Cardiometabolic Biomarkers

Beta-isomerized C-terminal telopeptide (a bone resorption marker) and procollagen 1 N-terminal propeptide (a bone formation marker) will be quantified using a Roche Hitachi Cobas e602 immunoassay analyzer, according to the manufacturer’s guidelines. Hormones (PTH), lipids, glucose and insulin, inflammation markers (C-reactive protein and serum IL-6), and potentially other cardiovascular or health markers will be analyzed according to standard hospital procedures.

##### Genotyping and Target Genetic Variants Analyses

We will target either candidate gene variants [48,49] or Genome-Wide Association–based variants previously related to skeletal muscle and bone health [50,51]. Genomic DNA will be extracted from residual blood samples from Becton Dickinson (BD) Vacutainer EDTA tubes using the MagSep Blood gDNA kit (0030 451.00, Eppendorf) or GeneJET Genomic Whole Blood DNA Purification Kit (#K0781 Thermo Scientific). Gene variants will be determined using the TaqMan SNP assay (Applied Biosystems, Thermo Fisher Scientific) by QuantStudio 7 Flex (Applied Biosystems, Thermo Fisher Scientific). Genotyping will be replicated in another independent institute, as previously described [52,53], to validate the results.

#### Muscle Function and Strength

##### Grip Strength and Gait Velocity

Grip strength will be measured using a hand dynamometer; a result of <20 kg for women and <30 kg for men will identify low muscle strength [54,55]. A 4 m gait velocity assessment will be performed by using the instrumented walkway, which has an acceleration (GAITRite), and by timing with a stopwatch, and reduced physical function will be determined as <80 cm/second. Both the grip strength and gait velocity thresholds noted are accepted as a measurement of sarcopenia [54,55] and will form the definition in this study.

##### Lower Limb Maximal Strength and LMQ

Participants will perform a one-repetition maximum (1RM) test on a leg press. This will be performed twice, with the first visit serving as familiarization. 1RM is defined as the heaviest weight lifted once with the proper technique and without compensatory movements [56]. Results from this study will guide appropriate prescription for the acute RE session.

LMQ, an estimate of specific force, has been shown to decrease with age and is described as the amount of force a muscle group can produce per unit of muscle mass [57]. We will calculate LMQ as follows:

LMQ = leg strength (kg)/(left leg lean mass [kg] + right leg lean mass [kg]) **(1)** [58].

Leg strength will be defined as the participants’ 1RM, and leg lean mass will be obtained from the DXA assessment.

##### Physical Performance Test

Participants will complete a physical performance test (PPT), adapted from Levinger et al [59], and will include 4 functional mobility tasks: (1) a gait velocity assessment (described earlier), (2) timed up and go test, (3) stair climbing power (SCP), and (4) stair descending. All tests will be scored in time (seconds).

The timed up and go test is a simple performance-based assessment that requires minimal equipment, including a standard arm chair (approximately 46 cm), a 3-m walkway with a floor mark, and a stopwatch (time, seconds). It is performed as time (seconds) taken to rise from a seated position, walk 3 m, turn, walk back to the chair, and then sit. The SCP consists of a rapid ascent of 10 stairs and is calculated as follows:

Power = force × velocity **(2)**

[60]

Velocity is calculated as the vertical distance of the stairs divided by the time it takes to ascend the stairs. Force is calculated as the participants’ body weight multiplied by acceleration due to gravity (9.8 m/s^2^). The stair descent will be the time to safely descend 10 stairs. The rest between ascent and descent will be 45 seconds. Participants will undergo 4 attempts on each task, and the best time will be recorded for each task. The PPT score will be the sum of the fastest times recorded for each test.

#### Aerobic Capacity and Vascular Health

Peak oxygen consumption will be assessed on a cycle ergometer with the initial intensity beginning at 10-30 W and increasing by 10-30 W×minute^-1^ according to participant ability. Participants will be monitored by a 12-lead electrocardiogram (Mortara, X-Scribe II). Oxygen consumption for each 15-second interval will be measured by gas exchange analysis (BreezeEx, version 3.02, Medical Graphics Corporation), with routine calibration of gas concentrations and flow before each test. The test will be terminated according to participants’ self-reported fatigue perception reaching a predetermined level (using the Borg scale, ratings of perceived exertion [RPE]=17) or clinical signs or symptoms [61]. Blood pressure will be monitored at baseline, regular intervals (each stage), and post exercise using a manual sphygmomanometer, and heart rate will be monitored via the 12-lead electrocardiogram.

Vascular endothelial function will be assessed by brachial artery flow–mediated dilatation, used in clinical trials as a reproducible method to assess endothelial function [62,63]. Vascular stiffness will be assessed by noninvasive measures of pulse wave velocity (simultaneous comparison of carotid and femoral arterial pulses) and pulse wave analysis (pulsations recorded at the brachial artery to produce central aortic pressure waveforms) using applanation tonometry (SphygmoCor EXCEL system V1, AtCor Medical) [64].

#### Questionnaires and Lifestyle Behaviors

##### Physical Activity Log

Participants will complete a lifestyle behavior and physical activity log. This log has been developed for the purpose of this study and will have questions related to average sleep cycles and normal physical activity levels on weekdays versus weekends (stratified into moderate, hard, and very hard activities). The physical activity component includes consideration for activities of daily living and structured exercise, with examples provided.

##### Dietary Behavior

A 3-day dietary log will be given to participants on their first visit, to be returned on visit 2 for investigators to analyze normal dietary behaviors. Participants are encouraged to eat normally while they are recording (ie, not to adjust food quantities) and are instructed to complete the dietary log on 2 weekdays and 1 weekend day (consecutively). This log also requests a timed record of physical activity behaviors, including the time and intensity, the time at which food and drinks are ingested, and the time and quantity of medications and supplements.

##### Falls Risk Questionnaire

The Falls Risk for Older People in the Community (FROP-Com) was developed by the National Ageing Research Institute as a modified version of the Falls Risk for Hospitalized Older People for better utility in the community [65]. The FROP-Com is simple, takes only 10-15 minutes to complete, is low cost, requires no equipment, and can be administered by any health professional. It is a comprehensive fall risk assessment, covering 13 risk factors for falls set out in 26 questions with dichotomous or ordinal scoring (from 0 to 3). The overall score is indicative of fall risk, with the total score ranging from 0 to 60, with higher scores indicating greater risk. The tool has demonstrated good reliability and has a moderate capacity to predict falls [65].

##### Mini Nutritional Assessment Questionnaire

The Mini Nutritional Assessment (MNA) is a widely used tool for assessing nutritional status in older adults. It is simple to administer, low cost, and validated, with high sensitivity, specificity, and reliability. The MNA classifies the interviewee as well nourished (score≥24), at risk of malnutrition (score between 17 and 23.5), or malnourished (score<17). The MNA also correlates with clinical assessments and objective measures, such as albumin, BMI, triceps skinfold, caloric intake, and vitamin status, and low scores are related to the incidence of clinical events and mortality [66-69].

##### Charlson Comorbidity Index Questionnaire

The Charlson Comorbidity Index (CCI) is a validated measure of 1-year mortality risk and burden of disease and is used in clinical research to understand the influence of comorbidities and predict outcomes [70-72]. In clinical practice, the CCI assists with the stratification of patients into subgroups based on disease severity to assist with targeted models of care and resource allocation. The CCI includes 17 comorbidities (with 2 subgroups for diabetes and liver disease) that are weighted from 1 to 6 for mortality risk and disease severity. These scores are then tallied to form the total CCI score.

#### Randomization

Following baseline assessments, participants will be randomized into the acute intervention to explore the characteristics of older adults (by sex) with low or high muscle function (Figure 1). Participants will be randomized individually by a researcher external to this project (they will have no contact with the participants before or during the trial). This person will also have no intellectual or personal investment in the study design, data collection, or outcome. The order of the 3 conditions for each participant (AER, RE, or control) will be randomized using a sealed envelope method (block allocation) to prevent carryover effects between conditions. The envelopes will be stored separately in a locked cabinet, and each envelope will contain 3 pieces of paper that will state “AER,” “RE,” and “CON.” These pieces of paper will be folded to reduce their transparency.

#### Study Intervention

##### Acute Intervention

Participants will complete the acute intervention (visit 3, 4, and 5) up to 14 days after completing the screening assessments and will complete the AER, RE, and CON conditions (Textbox 2) in a randomized order (described below). These visits will include blood sampling and optional skeletal muscle biopsies. Participants can elect to have none, 1 (at rest, for a baseline measure), or 4 biopsies (1 at rest for baseline and 1 following each condition). Each testing visit is approximately 3 hours in duration, including the 30-minute intervention (exercise or rest), and visits will be performed approximately 7 days apart, accounting for washout.

**Acute intervention.**

**Description of interventions**

- Aerobic exercise: Performed on the cycle ergometer for 30 minutes at an intensity corresponding to 70%-75% of peak heart rate; this is based on data obtained from the exercise capacity assessment. Intensity will be adjusted every 5 minutes to maintain the desired heart rate range.
- Resistance exercise: The protocol is as we have previously performed [10] and includes 30 minutes of strength and power exercises at intensities corresponding to 70%-75% of the predetermined one-maximal repetition based on the individual’s test results. Leg press will be performed as 5 sets of 10 rapidly concentric (as fast as possible) and slow eccentric (4 seconds) repetitions. Recovery between sets and exercise will be 2 minutes. Participants will also perform jumping sequences as 5 sets of 10 jumps (jumping as high as they can 10 times without stopping). Power training is effective to increase muscle strength and bone density and is safe for older adults [73-75].
- Control: This session will include 30 minutes of supine bed rest.

All testing visits will be monitored and supervised by accredited exercise physiologists (AEPs), who will follow the structured protocol as dictated for that particular session (AER, RE, and CON). The AEP will also monitor signs and symptoms in response to exercise training and will record Borg RPE, blood pressure, and heart rate at frequent time points. Any adverse signs and symptoms will be documented, including feelings of fatigue, soreness, light-headedness, and any injuries. Blood sampling and intravenous cannulation will be performed by personnel who are experienced in the technique, and muscle biopsy will be performed by an experienced medical physician.

##### Control Procedures

For testing visits 3, 4, and 5, participants will arrive at the laboratory between 7 AM and 8 AM following an overnight fast and with abstinence from exercise or reduced general activity (ie, heavy to moderate activities of daily living) in the preceding 24 hours and for all follow-up sessions. These sampling procedures will be followed at all visits to account for circadian or diurnal rhythms [76]. Participants may be requested to abstain from particular medications (eg, aspirin), as advised by the medical doctor, if electing for a muscle biopsy.

To assist with adherence to study protocols, participants will be monitored via regular communication with the study coordinator on the days preceding each study visit. As a general consideration for participation in this study, participants will be encouraged not to alter their current physical activity levels, exercise habits, or dietary intakes for the entirety of the study. Participants are asked to report whether there have been any alterations in medications throughout the study, as we request that all medication interventions are stable for at least more than three months.

##### Biospecimen Sampling Protocols

On arrival and following supine rest (approximately 15 minutes), a cannula will be inserted into the antecubital vein, and a baseline (resting) blood sample (40 mL; biopsy, if consented) will be obtained. These baseline (resting) samples will be obtained on the first visit only, serving as the baseline for all other visits thereafter. Four additional blood samples following the 30-minute acute intervention will be collected immediately after the intervention (0-minute time point) and at 30, 60, and 120 minutes postintervention (total of 100 mL) to observe changes in tOC, ucOC, COP, and other measures. If elected, a postintervention biopsy will be conducted at the 60 minutes time point. At all time points, blood samples will be collected into EDTA and serum-separating tubes vacutainers for the appropriate collection of serum or plasma. Following 10-minute clotting time, samples will be centrifuged for 10 minutes at 4° C and immediately transferred to long-term storage at −80° C in 2 mL aliquots for later analysis.

#### Outcome Measures

##### Primary Outcomes

The primary outcome for this study is the peak change in circulating levels of ucOC from baseline compared with postacute exercise blood sampling time points (0, 30, 60, and 120 minutes) following the 3 acute interventions (AER, RE, and CON) between the low muscle function and high muscle function groups.

##### Secondary Outcomes

The secondary outcomes for this study are (1) the difference in protein content related to atrophic and hypertrophic protein signaling at baseline between the low muscle function and high muscle function groups and (2) the difference in protein signaling (protein content) from baseline and compared with the postmuscle sampling timepoint (60 minutes) following the 3 acute interventions (AER, RE, and CON) between the low muscle function and high muscle function groups.

#### Data Collection and Analysis

##### Quantification of Osteocalcin

Serum tOC will be analyzed as described previously [9,10,19,39]. In brief, tOC will be measured using an automated immunoassay (Elecsys 170; Roche Diagnostics). Serum ucOC will be measured by the same immunoassay after absorption of cOC on 5 mg/mL hydroxyl-apatite slurry, following the method described by Gundberg et al [77].

##### Quantification of COP Cells

COP cell analysis will be performed as described previously [78,79]. In brief, peripheral blood samples (20 mL) will be collected (EDTA tubes) and processed for Ficoll-based gradient separation, and approximately 5×10^6^ peripheral blood mononuclear cells (PBMCs) will be obtained. Approximately 1×10^6^ PBMCs will be resuspended in fluorescence activated cell sorting (FACS) buffer, followed by a 10-minute blocking with fragment crystallizable receptor blocking reagent (BD Biosciences). Staining will then be performed with a viability marker (30 minutes), followed by washing (×2) with phosphate buffered saline (containing 5% fetal calf serum). Cells will be incubated with mouse antihuman CD45-Pacific Blue, CD3-PerCP, and CD19-APC (40 minutes). Staining of intracellular components will be permeabilized with Cytofix/Cytoperm (BD Biosciences) according to the manufacturer’s instructions, followed by incubation with mouse antihuman osteocalcin-phycoerytrhin at 4 °C (40 minutes), and then washed with Perm or wash buffer (×2).

##### Flow Cytometry

Cells will be analyzed using a BD FACS Canto II. FACS DiVa software will be used to analyze 50,000 total events for each sample and for the fluorescence minus one (FMO) controls. A total of 3 lasers and 8 different photomultiplier tube (PMT) channels will be used for the 6-color staining panel. Two FMOs will contain fluorochromes, except for the one to be controlled for. Compensation beads will be used to set the compensation controls for each fluorochrome. The PMT voltage values for fluorochromes will be set for each cell type based on the compensation controls. Doublet discrimination will be applied, and viability will be assessed by negative staining using the Live/Dead stain. Offline analysis will be performed using Flow Jo analytical software (Treestar).

##### Gating Strategy

Cells will be gated for size, shape, and granularity using forward and side scatter parameters, as previously described by our group [78,79]. Briefly, serial gating steps will be applied to quantitate cellular populations. First, dead cells will be excluded, and then, a region will be set to encompass lymphocyte-, monocyte-, and granulocyte-enriched areas, followed by doublet discrimination, T cell (CD3) and B cell (CD19) elimination. For COP cells, after gating on live single mononuclear cells (on forward and side scatter plots), the CD45 and osteocalcin double positive cells will be calculated. Cutoff points to assign antigen positivity will be performed against matching FMO controls. The use of FMO significantly increases the sensitivity and specificity of analysis, as they effectively minimize the effect of nonspecific antibody binding and cell-specific autofluorescence. The gating quantification will be performed twice for the accurate quantification of the percentage of COP cells.

#### Muscle Sampling Protocol and Analyses

##### Summary of Sampling Procedure

If elected, the muscle samples (1 or 4) will be taken from the vastus lateralis (approximately 150 mg) under local anesthesia (xylocaine 1%) by percutaneous needle biopsy technique, modified to include suction [80]. Excised tissue will be snap frozen in liquid nitrogen and stored at −80° C for later analysis. Proteins involved in muscle degradation and hypertrophy (ie, anabolic and catabolic pathways; ubiquitin-proteasome, autophagy-lysosome, and caspase-3–mediated proteolytic pathways) as well as glucose uptake will be assessed, as described previously [81-84].

##### Protein Extraction and Western Blotting

All muscle samples (baseline and postintervention samples) will be used to analyze the content and activation of signaling proteins involved in muscle degradation and hypertrophy by using western blotting, as described previously [84-86]. Western blotting is a method commonly used to detect and analyze the abundance and posttranslational modifications (such as phosphorylation) of proteins. Briefly, muscle samples will be homogenized in a radioimmunoprecipitation assay buffer using a TissueLyzer (QIAGEN). Then, proteins in the lysate will be separated based on protein molecular weight via gel electrophoresis. Proteins will be subsequently transferred to a polyvinylidene fluoride membrane where specific proteins can be probed using specific antibodies. Finally, signals generated through electrogenerated chemiluminescence will be detected and analyzed using ChemiDoc Imaging Systems (Bio-Rad Laboratories).

##### Primary Skeletal Muscle Cell Culture

A portion of the muscle obtained at rest (baseline) will be used for cell culture for future molecular analyses [87]. This will be established according to the method described by Blau and Webster [88] and by Gaster et al [89] and previously detailed by McAinch et al [90]. Briefly, muscle samples (50-100 mg) will be washed, minced, and enzymatically dissociated with trypsin. Cells will be cultured in a coated flask with extracellular matrix, and the growth medium (α-minimal essential medium [α-MEM]+10% fetal bovine serum+0.5% penicillin-streptomycin+0.5% antifungal) will be changed every other day until they reach 80% confluence. Then, satellite cells will be selected using CD56+ magnetic microbeads (Miltenyi Biotec) and transferred to bigger flasks coated with extracellular matrix to increase cell number (up to 4 passages). Once the cells reach 80% confluence, they will be differentiated using a differentiation medium (α-MEM+2% horse serum+0.5% penicillin-streptomycin+0.5% antifungal) for 5-6 days. Before the experimental treatment, cells will be starved for 1 hour in a serum-free medium (α-MEM+0.5% penicillin-streptomycin+0.5% antifungal).

In vitro treatment with ucOC at concentrations of 0 ng/mL, 30 ng/mL, and 100 ng/mL in serum-free medium for 60 minutes and 24 hours in the presence or absence of insulin (100 nM for the last 15 minutes) for the determination of glucose uptake (2-deoxy-D-[3H] glucose) and western blotting will be performed. Analysis of targeted proteins (described above) will be performed as described previously [39,91]. The dose response is important because the physiological effect of ucOC in muscles from mice and humans may be different, and the concentrations used are all physiologically relevant.

#### Participant Retention and Withdrawal

Once a participant is enrolled into the trial, the study coordinator will keep in contact with him or her for the entire study period. We estimate that the dropout rate in this population will be approximately 10%. Participants may withdraw from the study at any given time. The investigators or medical staff may also withdraw participants from the study due to safety or medical concerns.

#### Statistical Analysis and Determination of Sample Size

The primary endpoint for this study is the change in ucOC levels from baseline to the peak, postintervention sampling time point. The analysis will include a comparison of changes in ucOC levels in response to each intervention from baseline to postintervention sampling time points (0, 30, 60, and 120 minutes) between the low muscle function and normal or high muscle function groups, by using repeated measures analysis of variance (ANOVA). Comparisons of multiple means will be examined using a 2-factor (exercise type×time point) repeated measures ANOVA. For all significant interaction and main effects, a priori comparisons of means (baseline vs all postexercise time points) will be conducted using the Fisher least significant difference test (*P<*.05). Multivariable regression models will be used to determine associations between selected measurements, adjusting for BMI and sex. Data will be analyzed using the Statistical Package for the Social Sciences, version 22 (SPSS Inc), and statistical significance will be declared at *P*<.05.

In 10 postmenopausal women, we previously reported that the change in ucOC levels following exercise is approximately 9% [39]. We will recruit 54 participants (equal number of men and women) who will be dichotomized as low muscle function versus high muscle function (27 per group). After adjusting for a loss to follow-up rate of 10% (5.4/54), this sample size will be large enough to detect an estimated 4% difference in changes in ucOC levels (SD 6%) between groups with a type I error rate of 5%, type II error rate of 20%, and power of >80% (G*Power 3.1.9.2 for Windows) [92].

#### Data Monitoring

##### Data Management and Monitoring

Details of the procedures for data management have been reviewed and approved by the MH Human Research Ethics Committee and can be located via study reference, 2017/08. A trial management group (TMG) has been formed to manage potential risks and for structured oversight of the trial [93]. The type of oversight that this TMG will provide includes regular meetings to review individual safety reports and data relating to quality, protocol adherence, and participant retention rates. The TMG committee will include the principal investigator; individuals responsible for the daily running of the trial, including the trial coordinator; and an appointed independent member.

All electronic data will be stored on password-protected computers. Hard copies of any data will be kept in a locked filing cabinet in a secure office. All data collection tools and questionnaire data will be deidentified.

##### Harms

All adverse events associated with the study, or occurring during study participation, will be recorded. All adverse events will be reported to the TMG and ethics committees (MH and Victoria University) with strategies to reduce the risk for future events. The ethics committees have the power to pause or even stop the research in the case of a severe adverse event. The study personnel will monitor the clinical signs and symptoms of dyspnea, shortness of breath, nausea, faint-headedness and light-headedness, signs of inflammation, or infection throughout the study period.

##### Auditing

The TMG will meet and run an internal audit of the trial at regular intervals annually. The principal investigator, IL, is responsible for the overall conduct and preservation of the integrity of this trial and has extensive experience as a lead research investigator in numerous human clinical trials.

#### Ethics and Dissemination

This study and its protocols and data collection tools have been reviewed and approved by the MH Human Research Ethics Committee (ref approval number: HREC/17/MH/335; local project number: 2017/208), and local ethical approval has been confirmed at Victoria University as a mirror approval of MH. Any modifications to the study objectives, procedures, protocols, data collection tools, and study personnel will require a formal amendment from the ethics committee. All protocols related to consenting procedures, data collection and access to participant data, procedures for maintenance of participant confidentiality, and plans for dissemination of study results can be found in the reviewed and approved documents of trial reference 2017/208.

## Results

The trial is active, with participant recruitment and intervention delivery currently ongoing (MH Human Research Ethics Committee ref approval number: HREC/17/MH/335; Western Health Sunshine Hospital local project number: 2017/208; protocol version number: 6 25/06/2018). Recruitment for this trial began in February 2018, and 38 participants have completed the study as of May 26, 2019. The results of this study will be published throughout the trial, and the main study findings are expected to be published by June 2021.

## Discussion

Previous research suggests that crosstalk exists between the skeleton and skeletal muscle; however, this crosstalk has not been fully described or clearly elucidated, particularly in humans. In addition, a lack of physical activity accelerates the widespread cellular and molecular changes induced by aging, resulting in an increased prevalence of many chronic diseases [94]. Detecting the age-related conditions associated with inactivity and early intervention are essential for reducing the economic burden of aging on the health care systems worldwide. The development of affordable and universally accessible ways to prevent chronic disorders, such as tailored exercise programs, in combination with the development of robust blood biomarkers, will considerably improve the ability to predict and detect chronic diseases and reduce the health and economic burden caused by aging in a cost-effective manner [95].

This project is designed to uncover a novel crosstalk pathway between bone and muscle in older adults via ucOC. Current evidence from predominately cross-sectional studies suggests that osteocalcin, via ucOC, in humans may be associated with muscle function [10,19]. Evidence from animal and preclinical studies is encouraging, indicating a promising role for ucOC in improving muscle metabolism and function [16,17,25]. However, the roles of ucOC in humans and its relationship with muscle function and metabolism remain unknown. Evidence suggests that COP cells have a dynamic capacity to mobilize to sites of fracture repair and have the capacity to be upregulated under varying pathological or physiological bone forming processes, such as puberty and fracture [79,96-102]. However, it is unknown whether exercise stimulus, with osteogenic capacity, can increase the COP cell population and upregulate tOC and therefore ucOC.

The proposed project aims to overcome this gap by characterizing ucOC levels in older adults with a spectrum of muscle functions and in response to an acute exercise intervention. Importantly, we will investigate this bone and muscle crosstalk by determining the associations between the parameters of muscle function (ie, muscle signaling, muscle mass, and muscle strength) and ucOC. In the future, we plan to directly assess this association at a cellular level in human primary myotubes (those prepared in this study) to determine the direct effects of ucOC on muscle protein signaling and glucose uptake. The results of this study will provide a greater understanding of skeletal muscle metabolism and the crosstalk between muscle and bone in the older adult population. We aim to establish ucOC as a biomarker for muscle function and bone and muscle crosstalk in older adults to target potential mechanisms for future therapeutic studies. We also aim to advance the development of personalized clinical exercise guidelines for sarcopenia and other musculoskeletal conditions.

## Appendix

Multimedia Appendix 1 Schedule of enrollment, interventions, and assessments.

## Abbreviations

1RM: one-repetition maximum
AEP: accredited exercise physiologist
AER: aerobic exercise
AIMSS: Australian Institute for Musculoskeletal Science
ANOVA: analysis of variance
BD: Becton Dickinson
BMD: bone mineral density
CCI: Charlson Comorbidity Index
cOC: γ-carboxylated osteocalcin
COP: circulating osteoprogenitor
DXA: dual energy x-ray absorptiometry
FACS: fluorescence activated cell sorting
FMO: fluorescence minus one
FROP-Com: Falls Risk for Older People in the Community
IL-6: interleukin-6
LMQ: leg muscle quality
MH: Melbourne Health
MNA: Mini Nutritional Assessment
MSC: mesenchymal stem cell
PBMC: peripheral blood mononuclear cell
PMT: photomultiplier tube
PPT: physical performance test
pQCT: peripheral quantitative computed tomography
PTH: parathyroid hormone
RE: resistance exercise
RPE: ratings of perceived exertion
SCP: stair climbing power
TMG: trial management group
tOC: total osteocalcin
ucOC: undercarboxylated osteocalcin
α-MEM: α-minimal essential medium
