# Supplementary material for: Uncovering the Bone-Muscle Interaction and Its Implications for the Health and Function of Older Adults (the Wellderly Project): Protocol for a Randomized Controlled Crossover Trial
Source: JMIR Res Protoc. 2021 Apr 9;10(4):e18777. doi: 10.2196/18777 (PMC8065561; doi:10.2196/18777)
Supplement: Multimedia Appendix 1 [file resprot_v10i4e18777_app1.docx]

Multimedia Appendix 1. Schedule of enrolment, interventions, and assessments.

|  | **Study Period** | | | | |
| --- | --- | --- | --- | --- | --- |
|  | **Enrolment** | **Screening & Familiarisation** | **Study Interventions**  **(Acute Exercise)** | | |
| **Timepoint** (week)** | ***-t_1_*** | **Week 1 & 2** | ***Week 3*** | ***Week 4*** | ***Week 5*** |
| **Study Visit (number)** | ***-t_1_*** | **Visit 1 & 2** | ***Visit 3*** | ***Visit 4*** | ***Visit 5*** |
| **ENROLMENT:** |  |  |  |  |  |
| **Eligibility screen** | X |  |  |  |  |
| **Informed consent** | X | X |  |  |  |
| ***Medical History*** | X | X |  |  |  |
| **Random Allocation** |  | X |  |  |  |
| **INTERVENTIONS**  *Randomised controlled, crossover, performed in any order: |  |  |  |  |  |
| ***Acute Aerobic Exercise*** |  |  |  |  |  |
| ***Acute Resistance Exercise*** |  |  |  |  |  |
| ***Control Condition*** |  |  |  |  |  |
| **ASSESSMENTS:** |  |  |  |  |  |
| ***Bone density (DXA)***  ***Muscle strength***  ***Exercise capacity***  ***Functional measures***  ***Questionnaires*** |  | X |  |  |  |
| ***Blood sampling*** |  |  | X | X | X |
| ***Muscle sampling*** *(optional: 0, 1 or 4 muscle biopsies)* |  |  | X | X | X |
